# Certification as a Service

**DOI:** 10.1007/978-3-030-58858-8_21

**Published:** 2020-08-18

**Authors:** Sebastian Copei, Manuel Wickert, Albert Zündorf

**Affiliations:** 6 IT University of Copenhagen, Copenhagen, Denmark; 7 University of British Columbia, Vancouver, BC Canada; 8 Kassel University, Kassel, Germany; 9 Frauenhofer IEE, Kassel, Germany

**Keywords:** Microservices, Standardization, Certification, Agile

## Abstract

The development of industry 4.0 and smart energy IT-Components relies on highly standardized communication protocols to reach vendor-independent interoperability. In innovative and fast-changing environments, the support of standard protocols increases the time to market significantly. In the energy domain, the business models and the regulatory frameworks will be updated more often than the protocols. Thus agile development and supporting standardized protocols at the same time seems to be an issue. Here we will present a new proposal for standardization and certification processes as well as an architecture for a certification platform. Both will improve the support of agile development in the industry and energy domain.

## Motivation

In the energy and industry domain, vendor-independent scaling of distributed systems is a key challenge. To provide interoperability between different systems or integrated electronic devices (IED) the use of standardized communication protocols (such as OPC UA [11], IEC 61850 [8], IEC 60870-5-104 [7], etc.) is very common. While vendor independence is crucial for IEDs, which stay in operation for years or decades, for IED vendors itself selling certified products may also be a sales argument.

**Fig. 1.**
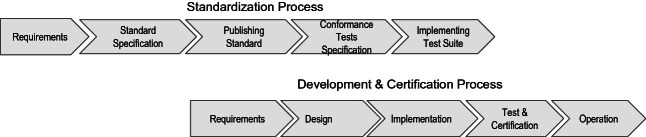
Classic standardization and product development processes

Figure 1 shows a classical standardization and certification scenario divided into two processes. The first (upper) process shows a view on developing a new version of a standard. The second process shows a classical waterfall model for applications where the certification is part of the testing phase. Note, both process views are very coarse overviews and do not provide a detailed look at a certain complex standardization or certification scenario.

The standardization typically begins with the specification of standard documents for a collection of requirements. Usually, the communication standard only describes the communication of a particular layer of the ISO/OSI communication stack. After publishing a finished version, conformance tests may be specified, and test suites may be implemented. E.g. The OPC Foundation offers a conformance test suite for its members [12]. The development of compliant products and the certification of them are illustrated as a classical waterfall model, where the certification is done in the test phase.

The key message of Fig. 1 is that the development process typically starts after (a new version of) the standard has been published. An earlier start of software development may result in incompatibilities with the standard. From a new requirement for the communication standard to a certified software version in operation, it may easily take several years. E.g., the Protocol IEC 61850-1 was published in 2003 in version 1.0 and 2013 in version 2.0. In the meantime, a lot of extensions were developed e.g., [1, 14]. From our practical experience with such communication standards, we see a lot of vendor-specific deviations. Therefore we assume that standardization approaches are often designed for classical and not for agile development processes.

Smart Energy Applications and Industry 4.0, are connecting classical industrial monitoring and control solutions with modern IoT-based technologies. Thereby modern software development processes are applied to address fast-changing requirements in both sectors to provide fast feedback cycles. Therefore we reconsidered how standardization and certification processes can be integrated into an agile product development process. It can be argued that stability is an essential requirement for communication protocols. But from our experience with more than 15 different projects, we see an upcoming preference for regular updates in operation over stability.

This paper presents two proposals to support agile standardization and certification processes. We propose a new standardization and certification process for communication protocols. For both process, proposals will use the terms standardization and certification. Our second proposal is an architecture for cloud-native certification services. The aim of the architecture is to support our idea of future agile certification processes. The proposals were developed with a background in smart energy systems and industry 4.0. However, our aim was to specify the process very generic to achieve transferability.

## Related Work

Agile standardization and certification processes have already been examined in various domains. Examples are high security system certification for aviation [4] and railways [2]. The authors of [4] present a way to certify security-critical components in a transportation system. They focus on high-level certificates. To provide the credibility of the certificates, the authors use a semi-formal description language. [2] shows a way to certify security-critical aerospace components. The authors use UML as a modeling tool to provide an incrementally changeable model description to achieve an agile certification process. However, the solutions presented in both papers are very domain-specific and focused on security certification. The given solutions only fit into their use cases and can not be used as a general approach. Furthermore, the solutions only cover the certification process on a client-side. Our solution wants to cover the whole process from developing a standard to certifying implementations of it.

An evolutionary standardization approach for file-based data is presented in [5]. The considered standardization focus is the engineering of automation systems. The basic idea is to start from an existing proprietary file format of one vendor and change it evolutionary to a neutral and later on to a common format, apparently often XML in that context. Similar to our process, this approach proposes a stepwise standardization. Nevertheless, the evolutionary approach is not intended to support agile development processes and focuses on file-based communication.

In [3] an agile standardization was performed for Process Control Equipment (PCE). The domain is close to the considered domain of this work. The authors require that standardization has to be done agile and “should proceed stepwise”. However, the focus of [3] is the concrete standardization of PCE Requests, not the standardization process itself.

## The Agile Standardization and Certification Processes

**Fig. 2.**
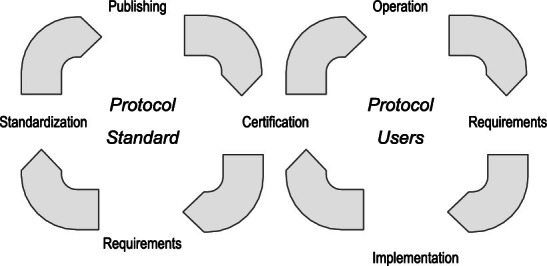
Agile standardization process

We propose an agile standardization and certification process that has two intertwined development cycles, cf. Fig. 2. As in other agile approaches, standardization and certification should be performed in small increments. The basic idea is to start with a minimal set of communication protocol features (e.g., establish a connection or login to a server) and add feature by feature in several iterations. Every iteration ends with a minor version change in the standard. The corresponding part of the overall standard is published e.g., via Github or some other configuration management service. Based on the publication of the standard for some features, the standard conformance tests that certify compliance with these features are extended or adapted and again published via a configuration management service. The standardization process runs iteratively, i.e., as soon as one feature has been completed, subsequent application development may start while the standardization continues with the next features.

The product development cycle, including the certification of a product, is shown on the right side of Fig. 2. The development of standard-compliant products may start with the requirements definitions for specific product features. The implementation of these product features may follow this. As soon as some feature is available, the feature implementation may try to pass the corresponding protocol conformance tests for a specific communication standard version. When the new product version is certified, it may be released and operated in production.

Each time a new version of the standard is exposed, and the corresponding conformance tests are deployed a test-driven development iteration of the products is ready to begin. Obviously, the conformance test will not be able to provide a complete test set for a product. However, these tests will support the product development relating to the communication interfaces. This approach has the advantage that first conformance tests will be available soon after the first iterations of the standardization process have completed. Thus, product development and standard development may be intertwined. Thereby, standard-compliant products will be available soon after the standard has reached a sufficient level of completeness. Besides, product development may provide feedback to the standardization process. Product development may e.g, point to overly complex conformance tests or inconvenient APIs or missing details, etc. This feedback may be used by the standardization process to enhance the standardization of the corresponding features and to come up with improved versions of the conformance tests. The importance of such feedback is also discussed in [5].

On the other hand, new versions of the standard lead to changes in the conformance test. This may result in failing tests for the new standard version and triggers the adaption of existing features. Such changes to already defined conformance tests may also happen when following features or later standardization iterations require previously standardized features to evolve. This is an infrequent problem inherent in agile software development. If a product development team wants to avoid such issues, it may wait until the standardization process has reached a sufficient level of completeness and stability. One can argue that this may be a drawback of our approach since stability is a critical requirement for communication devices in operation. However, since we have also to consider security for such field devices, we have to provide easy mechanisms to provide software updates in operation.

## Certification as a Service Architecture

For a certain standard, a certification service will support the agile standardization and certification process. Here we propose a microservice [6, 10] based certification as a service architecture. This architecture should support the understanding of our agile standardization and certification process on the one hand. An implementation of this architecture is currently work in progress and part of our future work.

**Fig. 3.**
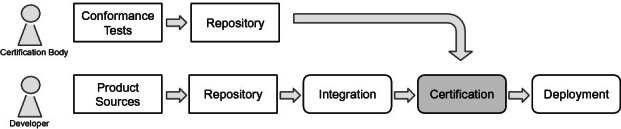
Continuous certification pipeline

Continuous integration and continuous deployment are methods to support fast feedback during agile development. A Certification as a service implementation extends a typical continuous deployment pipeline, as shown in Fig. 3. The certification step should be performed after the integration phase (which includes integration testing). The certification step consists of the execution of the conformance tests and the creation of a certificate. An implementation of our certification as a service platform will perform this step. This allows the deployment of certified products in every continuous deployment cycle. If conformance tests fail, the pipeline stops at the certification step, just like a failure during integration tests will stop the pipeline.

Each certification pipeline certifies a product according to a particular standard version. Whenever a new standard version is published, the respective conformance tests will be adapted or extended for this version of the standard. The certification bodies will add the standard to a repository. As soon as the new tests have uploaded, a product can be certified for the new standard version.

The certification service itself should be hosted as a service by the standardization or depending on organizational aspects, a certification body. As software as a service (SaaS), it should be compatible with a typical build pipeline software such as Jenkins. That allows an independent certification of products even with fast development cycles.

**Fig. 4.**
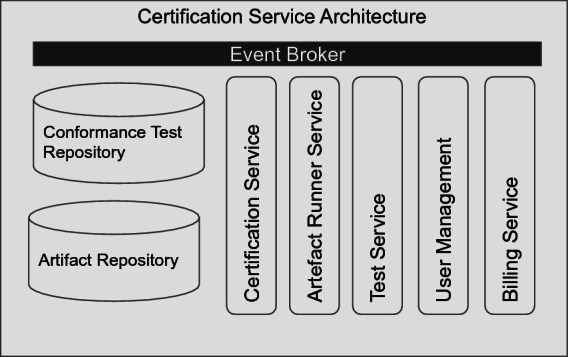
Certification service architecture

Our proposal for the certification service architecture is shown in Fig. 4. We defined five microservices, two repositories, and an event broker.

The repositories are responsible for storing a product for certification (artifact repository) and the conformance tests (conformance test repository). Both artifacts and conformance tests should be available in different versions. To perform the conformance tests, an instance of the artifact should be up and running for certification. The “Artifact runner Service” is responsible for running this artifact and configure it correctly. The “Test Service” will do the execution of the conformance tests. It will also provide test results for the “Certification Service”. The certification service will create a certificate for the artifact if all tests are passed successfully. The “User Management” and “Billing Service” have administrative responsibilities. Since the business model of a certification body is to issue certificates, it is necessary to implement user management and billing functionalities. The communication to the product development should be done by RESTful HTTP, to integrate with existing build pipelines easily. For internal communication, event sourcing should be used. Therefore we suggest making use of an event broker like Apache Kafka.

Our architecture aims to provide a proposal for certification as a service solution. Typical container orchestration tools can support implementations. Therefore an implementation of our service should be cloud-native [13].

## Conclusion and Future Work

We presented a new way to achieve a more agile process during the standardization and certification steps. We provide an architecture that should support the affected stakeholders during the whole process. On the one hand, this means that a standardization organization should have the possibility to provide fast incremental updates of their standards. On the other hand, we enable companies to use agile development processes for their certified implementation of standardized communication interfaces.

In the next steps, we will implement the architecture for a new communication standard for e-mobility use cases. We will examine how agile standardization approaches will work in that context. Furthermore, we will evaluate how this approach will support the agile development of prototypes for e-mobility use cases.


